# Vertical and horizontal environmental DNA (eDNA) patterns of fish in a shallow and well-mixed North Sea area

**DOI:** 10.1038/s41598-024-66912-2

**Published:** 2024-07-20

**Authors:** Nergiz Dukan, Isolde Cornelis, Sara Maes, Kris Hostens, Annelies De Backer, Sofie Derycke

**Affiliations:** 1 Flanders Research Institute for Agriculture, Fisheries and Food (ILVO), Ostend, Belgium; 2 Department of Biology, Marine Biology Section, Ghent University, Ghent, Belgium

**Keywords:** Ecological genetics, Biodiversity, Molecular ecology, Next-generation sequencing, Marine biology

## Abstract

The integration of eDNA metabarcoding into monitoring programs provides valuable information about fish community structures. Despite the growing body of evidence supporting the method's effectiveness in distinguishing fine-scale eDNA signals, there is a limited understanding of eDNA distribution in shallow, well-mixed environments, especially related to sampling depth. We analyzed 167 samples collected from the surface and bottom water at 17 locations of the Belgian Part of the North Sea (BPNS), where the deepest sampling point was 31 m, and compared this to beam trawl catch data collected simultaneously at the same locations. eDNA metabarcoding identified an additional 22 species compared to beam trawl catch data. Diversity measures and patterns were very similar between surface and bottom samples and revealed community patterns that were previously described by long-term beam trawl catch data. Surface and bottom samples had 39 fish species in common, while six and eight rare species were uniquely detected, respectively. Our results demonstrate that eDNA metabarcoding effectively identifies spatial community patterns of fishes in the highly dynamic environment of the BPNS regardless of sampling depth. Out of the six most common species tested, eDNA metabarcoding read abundances correlated strongly with catch-based abundance data for one species, but moderately for two others, indicating that inferring fish abundance and biomass via eDNA metabarcoding remains challenging.

## Introduction

As climate change, overfishing, eutrophication, and habitat disturbance alter species distributions and abundances, they persist as pressing challenges for the marine ecosystem. Consequently, biodiversity measurements and monitoring efforts have gained paramount importance^1,2^. Biodiversity surveillances can provide insights into various ecological concepts^3^, the status of the species^4^, and the effect of stressors on ecosystems^5^. Traditionally, biodiversity assessment has relied on methods such as catch-based surveys^6,7^, visual surveillance^8,9^, or acoustic surveys^10^. In the North Sea, beam trawl surveys are the most widely used technique for monitoring fish biodiversity. Unfortunately, these surveys have detrimental effects on benthic habitats leading to mortality of the benthos, physical disturbance of the seabed, and changes in biogeochemical characteristics due to the release of nutrients or contaminants associated with sediment^11^. Another inherent drawback of conventional monitoring procedures is their limited ability to detect rare and endangered species due to their infrequent occurrence^3,12,13^. Traditional methods also encounter difficulties in distinguishing physically similar species in their juvenile stages, species with phenotypical plasticity^14^, and generally detect invasive species only when they have already reached considerable abundances. The aforementioned limitations underscore the necessity for improvements in monitoring strategies and led to the incorporation of DNA metabarcoding to assess biodiversity using environmental DNA (eDNA) from aquatic environments^15–17^.

Environmental DNA (eDNA) refers to genetic material originating from organismal (derived from intact organisms) or extra-organismal (shed as metabolic waste or tissue from organisms, active propagules, or resulting from cell lysis) that is isolated from the environment^18^. Previous studies have demonstrated the effectiveness of eDNA metabarcoding in assessing diversity and spatial patterns of fish communities in the marine environment, particularly when combined with conventional surveillance methods such as visual or trawl surveys^19–22^. The approach has proven effective in describing spatial fish community structures with a fine-scale resolution, ranging from a few meters^23,24^ to a few kilometers (within 5 km)^25^, as well as over broader oceanographic distances (30 km)^19^. Even at highly dynamic shorelines with pronounced wave energy and tidal cycles, eDNA metabarcoding successfully distinguished spatial community patterns^24,26^. Several studies also differentiated eDNA signatures of fish communities across various depth gradients ranging from hundreds of meters^27^ to several meters^28,29^. However, these vertical patterns were observed in stratified open ocean waters or in areas where a pronounced halocline or thermocline is present; hence the eDNA dispersal between water masses was limited. For example, a study conducted at the Hywind Pilot Offshore Windfarm in Scotland, where the study area had a depth of approximately 110 m and a thermocline was detected at 20 m depth, revealed a vertical eDNA pattern of fish communities that correspond with their habitat preferences^30^. These studies demonstrate the strength of eDNA metabarcoding to determine community patterns for a wide range of spatial scales. However, they also reveal the understudied status of vertical eDNA patterns of fish communities in shallow and well-mixed coastal systems, which can provide crucial information for designing eDNA sampling schemes to improve biodiversity monitoring.

In addition to the fish community structure, quantitative measurements such as the abundance and biomass of fish populations play an essential role in assessing the status of both populations and communities. There is a growing body of evidence about the application of eDNA quantities as a proxy of actual fish abundance and biomass. Rourke et al.^31^ reviewed 63 research papers from both freshwater and marine eDNA studies and reported that 90% of those studies found a significant correlation between eDNA read counts and actual biomass. However, another meta-analysis suggested a weak quantitative relationship between biomass and metabarcoding sequences^32^. Some uncertainties associated with eDNA shedding, decay, and dispersal rates^33^, as well as the amplification biases^34^ may hamper the quantitative correlation between read counts and actual abundance/biomass. For example, no significant correlation was detected in a study that assessed the relationship between eDNA metabarcoding read count and the catch-based biomass of 11 species^35^. This reflects the ongoing challenge associated with utilizing the eDNA metabarcoding read abundance as an approximation for catch-based quantitative parameters.

The Belgian Part of the North Sea (BPNS) is located in the Southern Bight of the North Sea basin, covering a 3 454 km^2^ area. It is a shallow sea with an average water depth of 20 m and a maximum depth of 46 m, characterized by numerous sand bank systems that lie more or less parallel to the coastline^36,37^. Dominated by the tide-induced residual currents in the coastal areas and the English Channel waters entering through the Dover Strait, the area demonstrates high levels of turbidity due to advective transport and mixing^38^. Analyses of 540 beam trawl catches collected in the period 2008–2020 in the BPNS identified a coastal, offshore and transition fish community, the latter comprising a mixture of both coastal and offshore communities^39^. eDNA metabarcoding of water samples in 2021 was able to identify the same spatial (horizontal) distribution of fishes in the BPNS^40^. However, this study did not include locations from the western part of the transition and offshore zones.

In view of the vertical patterns observed in another shallow, wave exposed kelp forest ecosystem^41^ and the lack of western locations in the non-coastal zones of Cornelis et al.^40^, we designed this study to investigate the horizontal and vertical fish eDNA patterns in the BPNS. To this end, water samples were collected in autumn sampling campaign of 2022 from two depths (surface and bottom) at 12 locations and only from the bottom at 5 locations, encompassing the coastal, transition, and offshore zone. We used the 12S MiFish_U/E primers for metabarcoding, to target the species of Actinopterygii and Elasmobranchii simultaneously. We focus on the following research questions: (i) Is eDNA metabarcoding data able to reconstruct fish composition detected by the beam trawl catch data of the same sampling campaign? (ii) Does eDNA metabarcoding reflect the spatial fish community structure observed by long term monitoring trawl data? (iii) Is there a vertical fish eDNA pattern in the shallow, well-mixed BPNS that necessitates sampling at two depths? (iv) Do the read counts from eDNA metabarcoding correlate with the actual abundance and biomass of the most abundant fishes detected in the beam trawl catch data at the corresponding locations? The results allow to achieve a cost-efficient eDNA monitoring design of the area that accurately reflects beam trawl catch data.

## Materials and methods

### eDNA Sample collection

**Figure 1:**
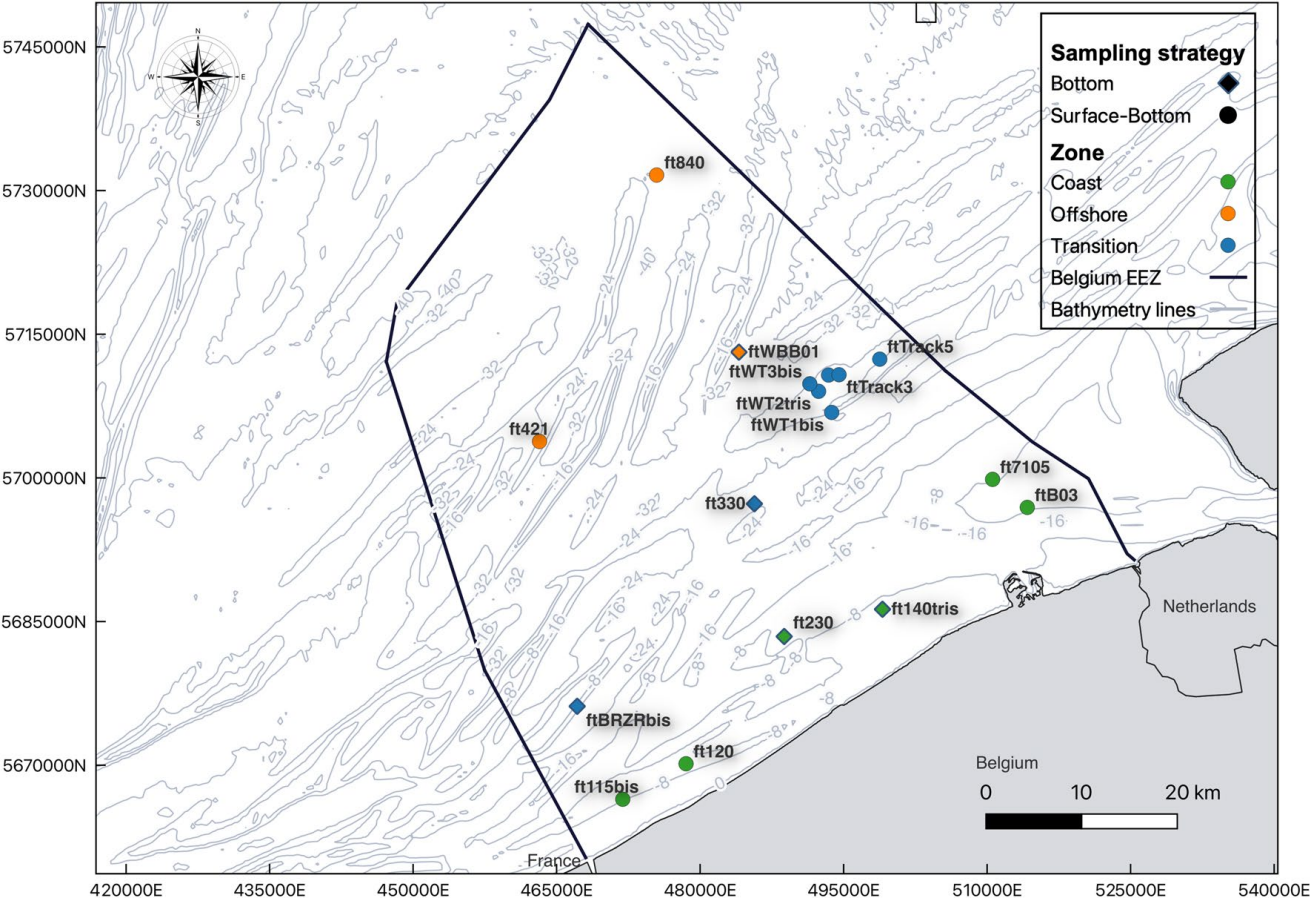
EDNA sampling locations in the Belgian Part of the North Sea. Dual sampling locations (surface and bottom) are represented by circles and bottom sampling locations are represented by diamond shapes. The colors of the location points correspond to the zone they belong to; coastal (green), transition (blue) and offshore (orange).

Sampling was carried out between September 26 and October 7, 2022 at the BPNS with the research vessel *Belgica*. Water samples were collected at 17 locations covering the distribution of the three distinct fish communities (coastal, transition and offshore) (Fig. 1). At twelve locations, water was sampled both at the surface and bottom to study the vertical distribution pattern of eDNA. A rosette equipped with 10 Niskin bottles, each with a 10 L volume was deployed to collect the water samples: bottom samples were taken 1 m above the seabed and surface samples 1 m below the sea surface. The rosette was held at the two depths for three minutes to allow the Niskin bottles to equilibrate with the surrounding water mass. To minimize the potential disturbance of eDNA caused by the downward movement of the rosette, the surface samples were collected before the bottom samples. Five biological replicates were collected at each depth per location, with each replicate corresponding to a single Niskin bottle. From each Niskin bottle, 2 L seawater was pre-filtered through a 200 µm sterilized nylon mesh to remove bigger particles and organisms that can potentially cause clogging during the subsequent filtering process. The pre-filtered water was then collected in commercial source water bottles, from which the source water was used to rinse the inside of the Niskin bottle before each deployment. Nine field controls were collected from rinsed Niskin bottles on nine distinct days to identify possible carry-over eDNA with the Niskin bottles between stations. Each time, a randomly selected Niskin bottle was sealed and then filled with 2 L commercial source water. The control sample was then collected by applying the same procedure as that followed for the biological samples. Water filtering was conducted onboard in a dedicated lab for eDNA analyses, right after the sample collection. The samples were filtered through a 0.45 µm Sterivex-HV filter (Millipore Sterivex^TM^, PVDF, with Luer outlet) using a peristaltic pump (Masterflex L/S® Variable Speed Pump HV-77910–75) until the filter was close to clogging. An average of 1.34 L was filtered per sample (± SD 0.68 L, max 2 L, min 0.15 L). Coastal samples tended to clog earlier than the offshore samples, as the concentration of suspended particles in the water column decreased with increasing distance from the coast (Supplementary Information 1; Filtered water volumes). Between locations, the pump was thoroughly rinsed with 10% bleach solution followed by commercial source water to avoid any potential cross-contamination between locations. Six filter negative samples were also collected using the same procedure by filtering 2 L of source water between. All filters were stored at − 20 °C until eDNA extraction.

### Trawl sampling and data processing

Following eDNA sampling, beam trawl samples were collected using an 8-m-long-beam-trawl equipped with a 22 mm mesh size cod-end and a bolder chain positioned ahead of the ground rope. The beam trawl was towed in the same direction as the current for 15 min at an average speed of 4 knots. The samples were then sorted and identified on board, mostly to species level. For each haul, the biomass and abundance of each species were recorded. The data was then cleaned only to include the stations that the water samples were also collected and the fish fraction of the total catch. The biomass and abundance values of the fish species were then standardized to a trawling distance of 1000 m^2^ (Supplementary Information 3).

### eDNA extraction

eDNA extractions were performed at Flander Research Institute for Agriculture, Fisheries and Food (ILVO), in a dedicated pre-PCR laboratory only used for eDNA analyses, following a slightly modified version of the standard extraction protocol of the DNeasy Blood&Tissue kit (Qiagen). The filters were lysed overnight with 800 µl lysis buffer (718 µl ATL (Qiagen) + 2 µl IPC (1/10 000) (gBlocks® Fragments, Integrated DNA Technologies) + 80 µl proteinase K (Qiagen)) at 56 °C in an incubator (Incubator-Genie, Scientific Industries). After the incubation, 750 µl of the lysis buffer was transferred to 1.5 ml LoBind tubes (Eppendorf). Purified eDNA was eluted with 2*50 µl 1 × TE buffer (70 °C). Additionally, a negative DNA extraction control was included by applying the same procedure to a blanco Sterivex filter. All extracts were stored at − 20 °C until further processing.

### Library preparation and sequencing

PCR amplification was performed using uniquely tagged MiFish primers (Supplementary Information 1; Primer tags) targeting the hypervariable region (163–185 bp) of the 12S rDNA mitochondrial gene^16^. A minor modification was introduced whereby the universal forward and reverse MiFish_U primers were degenerated to amplify both Actinopterygii (bony fishes) and Elasmobranchii (cartilaginous fishes) (MiFish_U/E_F: 5’-GTYGGTAAAWCTCGTGCCAGC-3’ and MiFish_U/E_R: 5’CATAGTGGGGTATCTAATCCYAGTTTG-3’). PCR was prepared in a 25 µl volume in triplicates for each sample, which included 12.5 μl KAPA HiFi Hotstart 2 × ReadyMix (Roche), 0.5 μl Bovine Serum Albumin (BSA) (10 mg/μl), 1 μl of each uniquely tagged reverse and forward primer (2.5 μM), 7 μl UltraPure™ water (UltraPure™ DNase/RNase-Free Distilled Water, Invitrogen™) and 3 μl extracted eDNA. Six PCR negative controls were also included by adding 3 μl UltraPure™ water to the mixture instead of extracted eDNA. The preparation step was performed on ice to prevent primer degradation. PCR amplification was performed on a Bio-Rad T100™ thermal cycler in 96-well PCR plates and conducted with an initial denaturation (95°C for 3 min) followed by 40 cycles of denaturation (98 °C for 20 s), annealing (62 °C for 15 s) and extension (72 °C for 15 s), and finalized by a final extension (72°C for 5 min). A random subset of PCR products from each PCR replicate was selected to be analyzed for 12S amplification efficiency using the Bioanalyzer (2100 Bioanalyzer Instrument, Agilent), following the standard protocol of the Agilent DNA 7500 Kit. In total, 167 samples (145 biological samples and 22 negative controls) were amplified. The products were pooled per PCR replicate in a 2 ml LoBind tube (Eppendorf) by taking 5 µl from each well. Next, the three PCR pools were purified with CleanNGS beads (CleanNA) according to the manufacturer’s guidelines. Briefly, the samples were magnetized with 1 × the sample volume of CleanNGS reagent and rinsed twice with 80% ethanol. Purified libraries were then eluted with 100 µl Tris–HCl buffer (10 µM, [ph 8.5]). Next Generation Sequencing of the 12S rDNA was conducted at Admera Health Biopharma Services (New York, US). Prior to sequencing, the libraries were adaptor-ligated and equimolar concentrations were prepared by the service provider. The pooled libraries were then sequenced using the Illumina MiSeq platform with 15% PhiX control using V3 chemistry for paired-end sequencing (2 × 300 bp).

### Bioinformatics

The raw sequencing reads were quality checked with FASTQC v0.11.9^42^ and the read counts were calculated to verify that forward and reverse reads were equal for each pool. Subsequent bioinformatics steps were performed using the slightly adapted version of meta-fish-pipe v1.0 bioinformatics module, as described by Collins et al.^43^. The initial step of this module was carried out using cutadapt v2.3^44^, which involves reorientation, demultiplexing, and removal of the primers and barcode tags. Specifically, the sequences were grouped according to their sense and antisense orientation, and those that did not contain both primers in their 3’ and 5’ ends were discarded with a maximum error rate of 15%. This specific error rate was implemented due to the use of degenerated primers, allowing maximum mismatch of three bases in the forward primer, and four bases in the reverse primer. Next, reoriented sequences were demultiplexed based on their unique barcode tags, with a maximum error rate of zero to prevent demultiplexing errors. Finally, the tags and primers were removed from the demultiplexed reads and the reads with a length shorter than 105 bp were discarded. Following demultiplexing, sequences were further processed using DADA2 v1.10.1^45^. The steps involved are quality trimming, denoising, dereplication, merging, removing chimeras, and creating Amplicon Sequence Variants (ASVs).

Taxonomic assignment was initially conducted based on the custom-made reference database, which was previously created according to the detected fish species in beam trawl sampling campaigns of ILVO in the BPNS. The database consists of complete or partial 12S reference sequences of 122 fish species. Twelve of these sequences were obtained via Sanger sequencing from tissue samples, while the remainder were compiled from the NCBI dataset. The ASVs were initially compared against the custom reference database in R v 3.6.3^46^ using the DADA2 package (“assignTaxonomy” function, minimum bootstrap = 80)^45^. The unassigned ASVs were subjected to a further query against the custom reference database and NCBI database using BLAST + v2.12.0^47^ with a query coverage high-scoring segment pair percentage (qcovHSP) threshold of 75% and identity percentage threshold of 97% (accession date: 23/03/2023). Unaccepted names were corrected for accepted scientific names using WoRMS Taxon Match Tool^48^. Finally, the species-level assignments of *Eutrigla gurnardus, Chelidonichthys lucerna, Chelidonichthys cuculus,* and *Chelidonichthys spinosus* were grouped at family level as Triglidae sp., given that these species have identical 12S marker sequences. Likewise, *Hyperoplus lanceolatus, Hyperoplus immaculatus, Ammodytes marinus,* and *Ammodytes tobianus* were grouped as Ammodytidae sp. *Alosa alosa*, *Alosa fallax*, *Chelon ramada,* and *Chelon auratus* were grouped at genus level. Contaminant ASVs were identified and removed from the dataset using the MICRODECON package v 1.0.2^49^ with the primary function “decon” and the default settings by grouping samples according to their location. This method works with the principle of identifying the “constant” contaminants in control samples and calculates the read counts originated from the contamination, subsequently subtracting those reads from the biological samples. According to that, some contaminant ASVs were completely removed from the dataset while some ASVs were quantitatively adjusted. The PCR replicates were then concatenated per sample by summing the read counts. Seven samples that have yielded no sequences at all were also automatically excluded in the taxonomic assignment step.

### Data analysis

All downstream analyses were performed using the VEGAN package v 2.6.4 in R^50^. The locations were categorized into coastal (6 locations), transition (8 locations) and offshore (3 locations). Stacked bar plots were made to visualize the read counts and number of ASVs assigned to fish, human, other taxa and unassigned reads/ASVs in each sample (Supplementary Information 4; Figures S1 & S2). Pie charts were generated at the kingdom, class and family level to depict the proportion of total read counts of various taxa. The data was then filtered to include only Actinopterygii, Elasmobranchii and Chondrichthyes species. All detected freshwater species (*Alburnus alburnus, Barbatula barbatula, Gobi gobio, Rutilus rutilus, Squalius cephalus, Oreochromis niloticus,* and *Cyprinus carpio haematopterus x Megalobrama amblycephala)* were excluded from the dataset since our study focuses on the marine ecosystem.

A Venn diagram was constructed with the presence/absence data of the species using the VENNDIAGRAM package in R^51^ to compare fish species obtained from the simultaneously collected eDNA surface samples, eDNA bottom samples, and beam trawl samples. For the alpha diversity analysis, we implemented coverage-based rarefaction to ensure the equal completeness of the samples for diversity comparison^52^. To do that, the coverage values of the samples were first calculated at fish ASV level using “phyloseq_coverage” function METAGMISC package v 0.0.4^53^. The samples were then rarefied to 0.983 coverage value using “phyloseq_coverage_raref” function as this value was observed to be the closest value to the minimum coverage for the majority of the samples. The ASVs were then aggregated to the species level to obtain the community data. To test for differences in species richness (S) and Shannon diversity index (H’) of eDNA data per depth (surface or bottom) across fish community zones (coastal, transition, and offshore), a two-way Analysis of Variance (ANOVA) including main factors ‘zone’ (with 3 levels: coastal, transition and offshore) and ‘sampling depth’ (2 levels: surface and bottom) and their interaction was performed. The assumptions for the linear models were assessed through diagnostic plots for each statistical analysis. Specifically, normal QQ plots with a 95% confidence interval comparing theoretical quantiles and standardized residuals were used to test for normality. For evaluating the homoscedasticity, we analyzed the scatters of the square root of standard residuals against fitted values (Supplementary Information 4; Figure S4). We used robust linear model instead of a linear model for Shannon diversity index, since the normality assumption were not met for that measurement. Post-hoc Tukey HSD (Honestly Significant Difference) tests were conducted for the significant main effects.

For beta diversity analysis, we first aggregated the unrarefied fish ASV table to species level and then standardized the unrarefied community data by implementing “eDNA index” method based on Wisconsin double transformation^54^. Briefly, eDNA read count proportions were calculated, followed by the scaling the abundances from 1 to 0 based on the species with the highest eDNA proportion. Beta diversity was then calculated based on a Bray–Curtis dissimilarity matrix and a Non-metric Multidimensional Scaling (NMDS) plot was used to visualize the ordination of the data grouped by sampling depth and zones. The statistical significance of these two main factors (depth and zone) and their interaction (depth*zone) on community composition were tested with permutational multivariate analysis of variance (PERMANOVA) in R (VEGAN package, “adonis2” function; permutations = 9999), followed by pairwise multilevel comparison (PAIRWISEADONIS package, “pairwise.adonis2” function; permutations = 9999, correction method = Benjamini & Hochberg)^55^. We also performed multivariate homogeneity of group dispersions test (BETADISPER) to assess the influence of dispersion on the statistical significance obtained (VEGAN package, “betadisper” function; type = centroid). An Indicator Species Analysis (INDISPECIES package, “multipatt” function; permutations = 9999, cluster = Zone, duleg = TRUE)^56^ was performed both for eDNA metabarcoding and beam trawl data to assess which method more accurately reveals the indicator species associated with the zones when compared to the long-term monitoring data. A threshold of 0.7 or higher for the indicator value was chosen to classify a species as a good indicator of the corresponding zone. A heatmap was also generated based on the indexed community data to visualize the association of the species with the zones.

We applied Spearman’s correlation coefficient to the most abundant species to assess the correlation between eDNA read abundance and catch-based abundance/biomass. eDNA sample replicates were averaged per station in the indexed dataset. *Limanda limanda, Pleuronectes platessa, Solea solea, Echiichthys vipera, Merlangius merlangus*, and *Trachurus trachurus* were selected based on their high read abundance across most locations, and their detection in the beam trawl data in at least ten locations. Spearman’s correlation coefficient were calculated to evaluate the relationship between these species’ eDNA indices and relative catch-based abundance/biomass using the GGPUBR package in R (“stat_cor” function, method = “spearman”)^57^.

## Results

### Sequence processing and taxonomic assignment

In total, the Illumina sequencing generated 16 474 726 raw paired-end reads from three libraries and 167 samples. After the removal of the reads without primers and the demultiplexing steps, a total of 12 480 536 (75%) paired reads were retained for DADA2 (Supplementary Information 1; DADA2 track). For the 145 biological samples, the average number of sequence reads was 43 862 (± SD 55 622). This resulted in an average of 740 ASVs per sample (± SD 580). Out of 6836 ASVs, 48% (or 3262) were assigned at the species level (Supplementary Information 2; Raw ASV table). The kingdom Animalia accounted for the highest percentage read counts of all assigned ASVs (38%), with a total of 2,300,504 reads, followed by Chromista with 27% (1,629,486 reads), and Bacteria with 23% (1,414,524 reads). The kingdom Animalia comprised 438 unique ASVs, of which 354 ASVs belonged to cartilaginous and bony fishes with a total sequence read count of 2,160,634 (94% of all Animalia reads). These ASVs corresponded to 26 orders, 41 families, 61 genera, and 68 species (*Alosa* sp., Ammodytidae sp. and Triglidae sp. were assigned to genus and family level, respectively) in the raw eDNA data. Actinopteri taxa represented 99.5% of all fish read counts (2 150 453 reads) with 340 unique ASVs. The families with the highest relative read counts were Clupeidae (32.3%), Pleuronectidae (17.3%), Engraulidae (9.9%), Soleidae (8.4%), Ammodytidae (6.2%) and Gobiidae (5.5%) (Supplementary Information 4; Figure S3). The Elasmobranchii represented only 0.5% of the total read counts distributed across six species: *Dasyatis pastinaca*, *Scyliorhinus canicula*, *Mustelus asterias*, *Raja clavata*, *Raja brachyura* and *Alopias vulpinus*.

dataset.

MicroDecon identified 887 ASVs as contaminants, 391 of them were totally removed from the dataset. The removed ASVs includes 30 fish ASVs corresponding to 13 marine fish species. Decontamination led to the exclusion of six species from further analysis, namely *Zeus Faber, Raja clavata, Scyliorhinus canicula, Microstomus kitt*, *Callianymus lyra* and *Spondyliosoma cantharus.* After removing all ASVs belonged to non-target taxa and fresh water fish species, the marine fish ASV dataset comprised 138 samples and 314 ASVs (Supplementary Information 2; Post-MicroDecon marine fish ASVs).

### Fish detection in trawl catches versus eDNA from surface and bottom water

**Figure 2:**
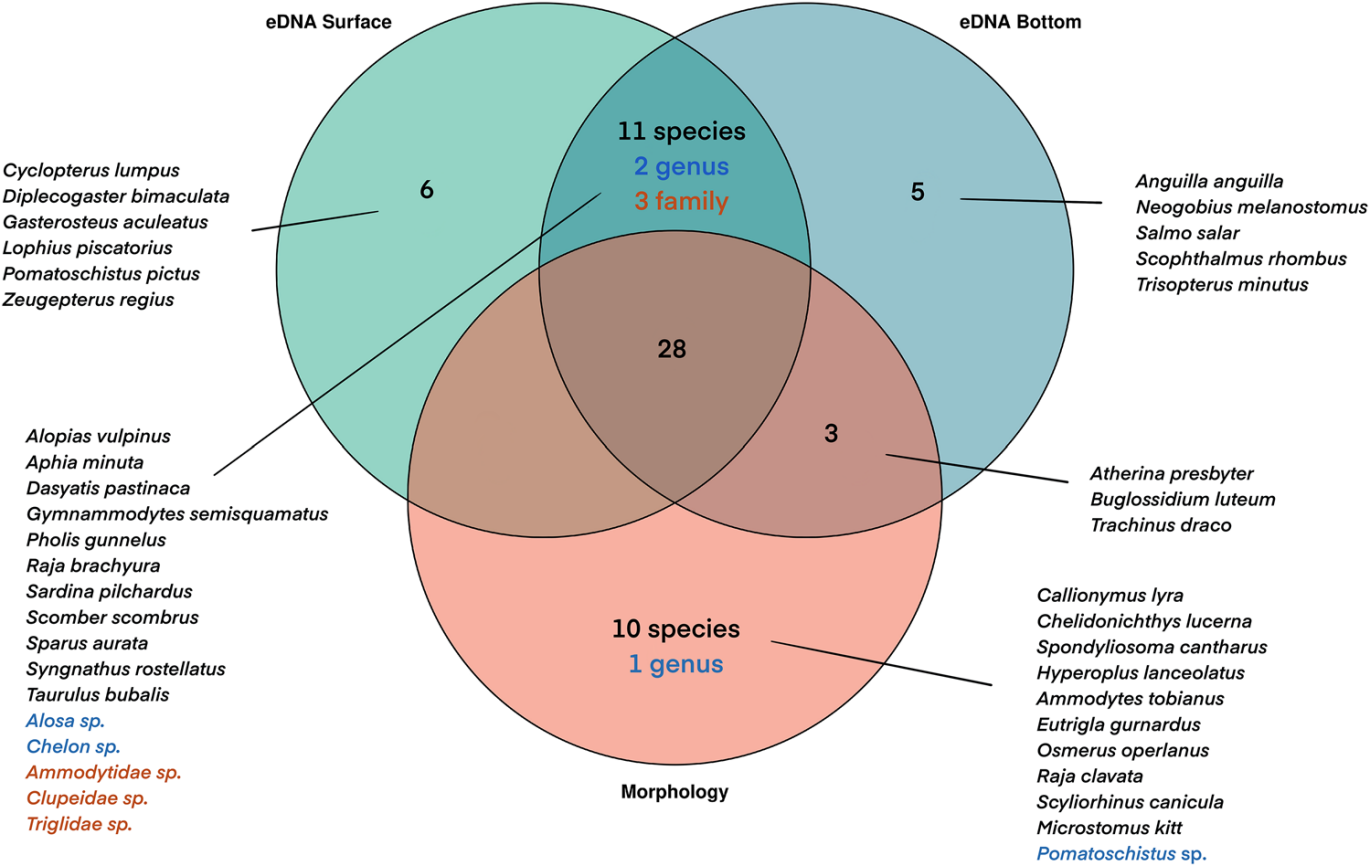
Venn diagram of the species based on the detection method. The full list of the species including all detection intersects is given in Supplementary Information 2; Species detection list.

eDNA dataset used for statistical analyses included 53 species level, three family level (Triglidae sp., Clupeidae sp. and Ammodytidae sp.) and two genus level (*Alosa* sp. and *Chelon* sp.) identification of marine fish taxa for further analyses. On the other hand, 41 fish species and one genus were morphologically identified from trawl catches, while 45 and 47 species were detected with eDNA from surface and bottom samples, respectively (Fig. 2). Most species detected in the surface samples were also detected in the bottom eDNA samples (39 out of 53 species), while respectively 28 and 31 species were shared with the beam trawl samples (Supplementary Information 2; Species detection list). Six and eight species were detected only in the eDNA surface and bottom samples, respectively, and these species were found in maximum two samples with the exceptions of *Trachinus draco* and *Scophthalmus rhombus *which were found in three and four samples, respectively. Conversely, 10 species were exclusively identified through morphological identification. *Ammodytes tobianus* and *Hyperoplus lanceolatus* on one hand, and *Eutrigla gurnardus* and *Chelidonchthys lucerna* on the other hand have identical 12S sequences and were therefore not identified through eDNA metabarcoding. *Osmerus eperlanus* and *Spondyliosoma cantharus* were detected in one location in very low abundances with the beam trawl. *Callionymus lyra*, *Raja clavata*, *Scyliorhinus canicula* and *Microstomus kitt*, which were detected also in very low abundances in maximum three locations, were also detected with eDNA metabarcoding initially, but excluded from the dataset after decontamination. *Callionuymus lyra*, on the other hand, was detected by the trawl data in relatively moderate abundances but also excluded from the dataset after decontamination. The morphological identification of some specimens of *Pomatoschistus* sp. was limited to the genus level due to their similar morphological characteristics. However, eDNA metabarcoding successfully distinguished between three *Pomatoschistus* species: *Pomatoschistus pictus, Pomatoschistus lozanoi,* and *Pomatoschistus minutus*.

### Alpha diversity patterns from eDNA between depths and zones

**Figure 3:**
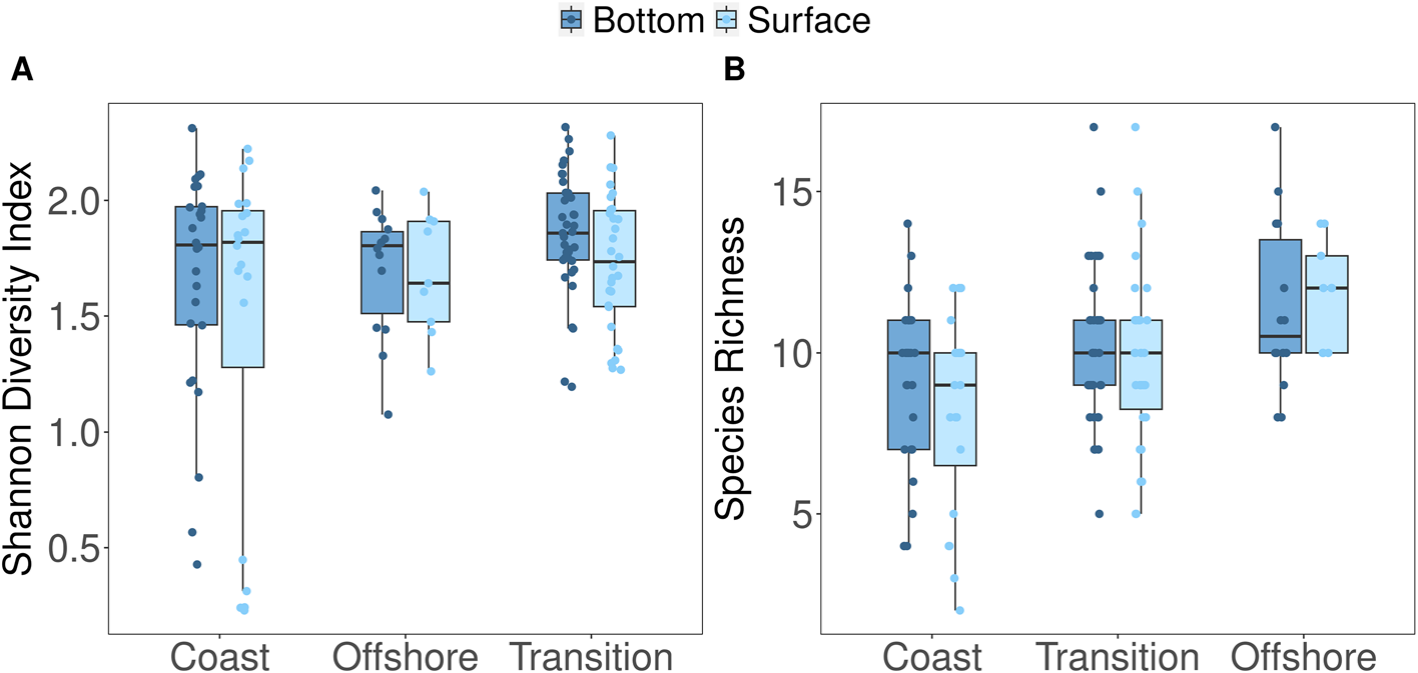
Box plots for alpha diversity measurements per zone comparing eDNA surface and bottom samples. A) Shannon diversity index B) Species richness.

The ANOVA results for the species richness performed after fitting a linear model revealed no significant interaction of the factors zone and depth. The richness was not significantly different between the two depths (*p* > 0.05), but showed significant differences between the zones (p < 0.001) (Fig. 3). Post hoc analysis revealed that all zones were significantly different from each other for species richness, with the strongest difference being between coast and offshore zones (p < 0.0001). The Shannon diversity index with robust linear model, on the other hand, did not show any significance for the interaction and both main factors (Supplementary Information 4, Table S1 & S2).

### Community composition catterns from eDNA between zones and depths

**Figure 4:**
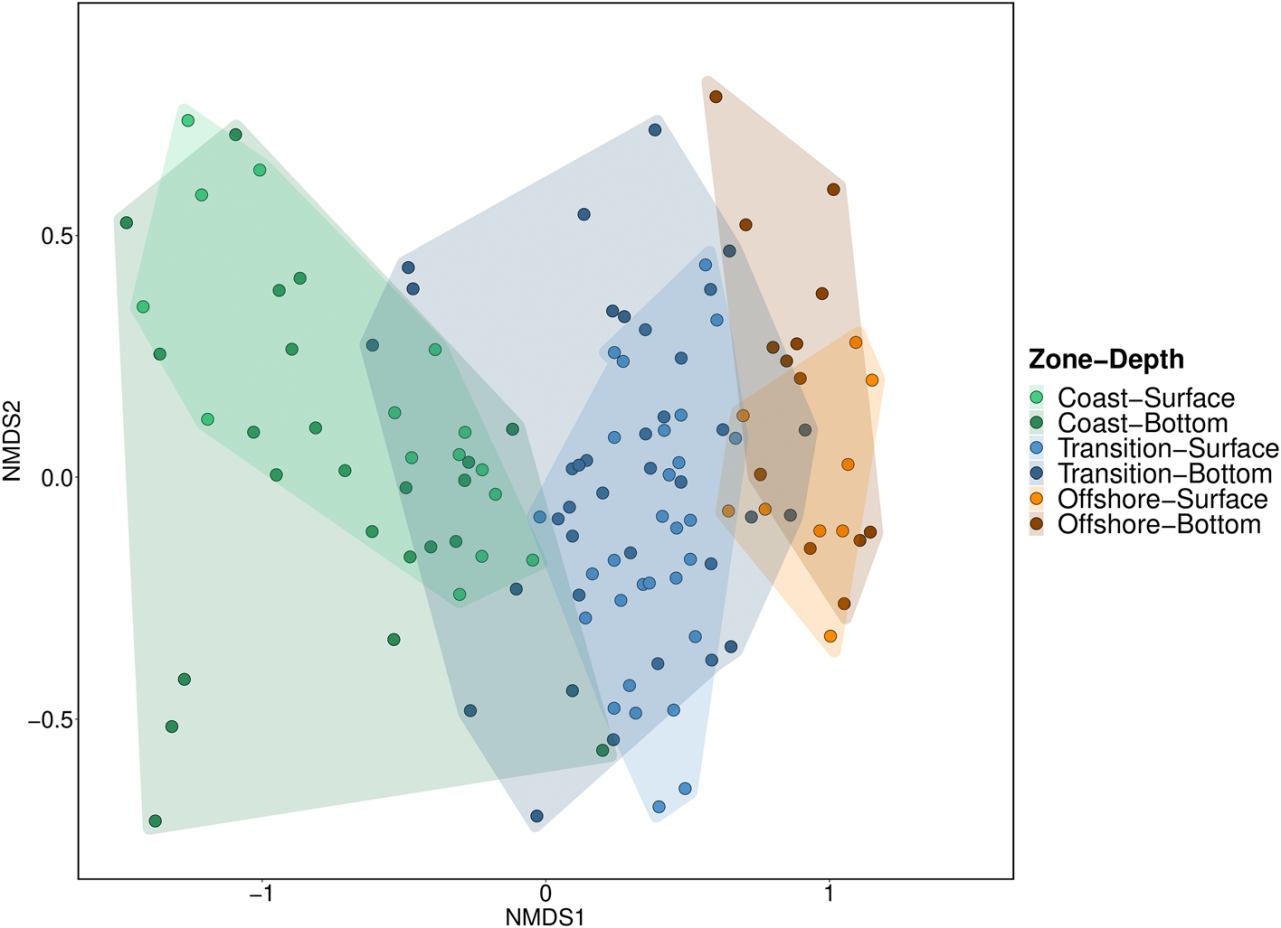
Non-parametric Multidimensional Scaling (NMDS) plot for the fish community (Stress value = 0.1654). Groups are defined for both vertical and horizontal space.

Community composition was not significantly affected by the interaction effect of depth* zone (pseudo-F = 0.21, *p* = 0.99). Factor depth had borderline significance on community patterns (pseudo-F = 2.56, *p* = 0.0497). Zone, however, had a strong significant effect on the community composition (pseudo-F = 46.98, *p* = 0.0001). Post hoc pairwise analysis showed that communities of all three zones (coastal, transition, and offshore) were significantly different (*p* = 0.0001) (Supplementary Information 4; Table S3 & S4). The Non-parametric Multidimensional Scaling plot discerned clear clustering of the coastal, transition and offshore zones, whereas the depth groups overlapped each other (Fig. 4). Betadisper yielded statistical significance for the factor zone (*p* = 0.012), but not for depth factor (*p* = 0.12). However, the plot still supported the distinct separation of the zones and overlap of the depths, suggesting that the significant zone effect was probably a combination of location and dispersion effects (Supplementary Information 4; Figure S5).

The heatmap revealed that 18 species had relatively high read abundances compared to the others and illustrated fish species that were more abundantly detected in coastal locations (*Pomatoschistus minutus, Pomatoschistus lozanai, Clupea harengus,* and *Platichthys flesus*), those that were more prevalent in transition and offshore locations (*Scomber scombrus, Trachurus trachurus,* and *Trisopterus luscus*) and those that were widely distributed across the BPNS (Supplementary Information 4; Figure S6). *Sardina pilchardus* has emerged as the species with the highest read count in total, exhibiting notably high read abundances in transition/offshore areas and western coastal locations, despite not being detected in trawl data.

Indicator Species Analysis for the eDNA metabarcoding data demonstrated a strong association of *Limanda limanda, Sprattus sprattus, Clupea harengus, Platichthys flesus, Pomatoschistus lozanoi* and *Pomatoschistus minutus* with the coastal zone, whereas *Mullus surmelutus, Scomber scombrus, Trachurus trachurus, Echiichthys vipera* and Ammodytidae sp. found to be the best indicators for the offshore zone. The only species that showed association with the transition zone was *Engraulis encrasicolus*. On the other hand, beam trawl catch data strongly associated *Merlangius merlangus*, *Myoxocephalus scorpius* and *Solea solea* with the coastal zone, in addition to the species determined by eDNA metabarcoding. *Pegusa lascarsis* was observed to be the indicator species of the offshore zone, whereas no association was observed for the transition zone. (Supplementary Information 4; Table S5 & S6).

### Quantitative assessment of eDNA read counts

**Figure 5:**
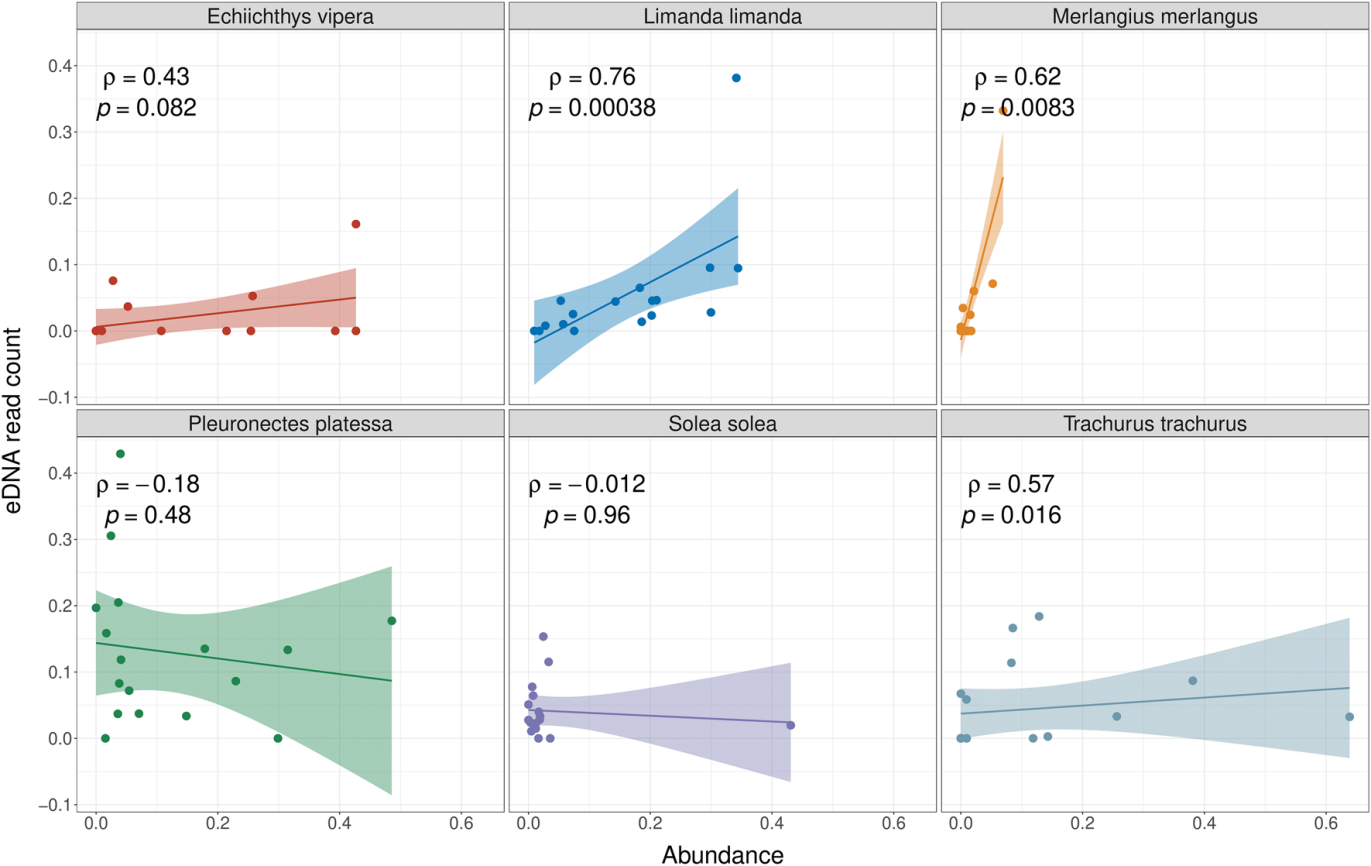
Spearman’s correlation coefficient plots contrasting indexed read counts and relative catch-based abundance. Shaded area around the linear regression line represents the 95% confidence interval.

**Figure 6:**
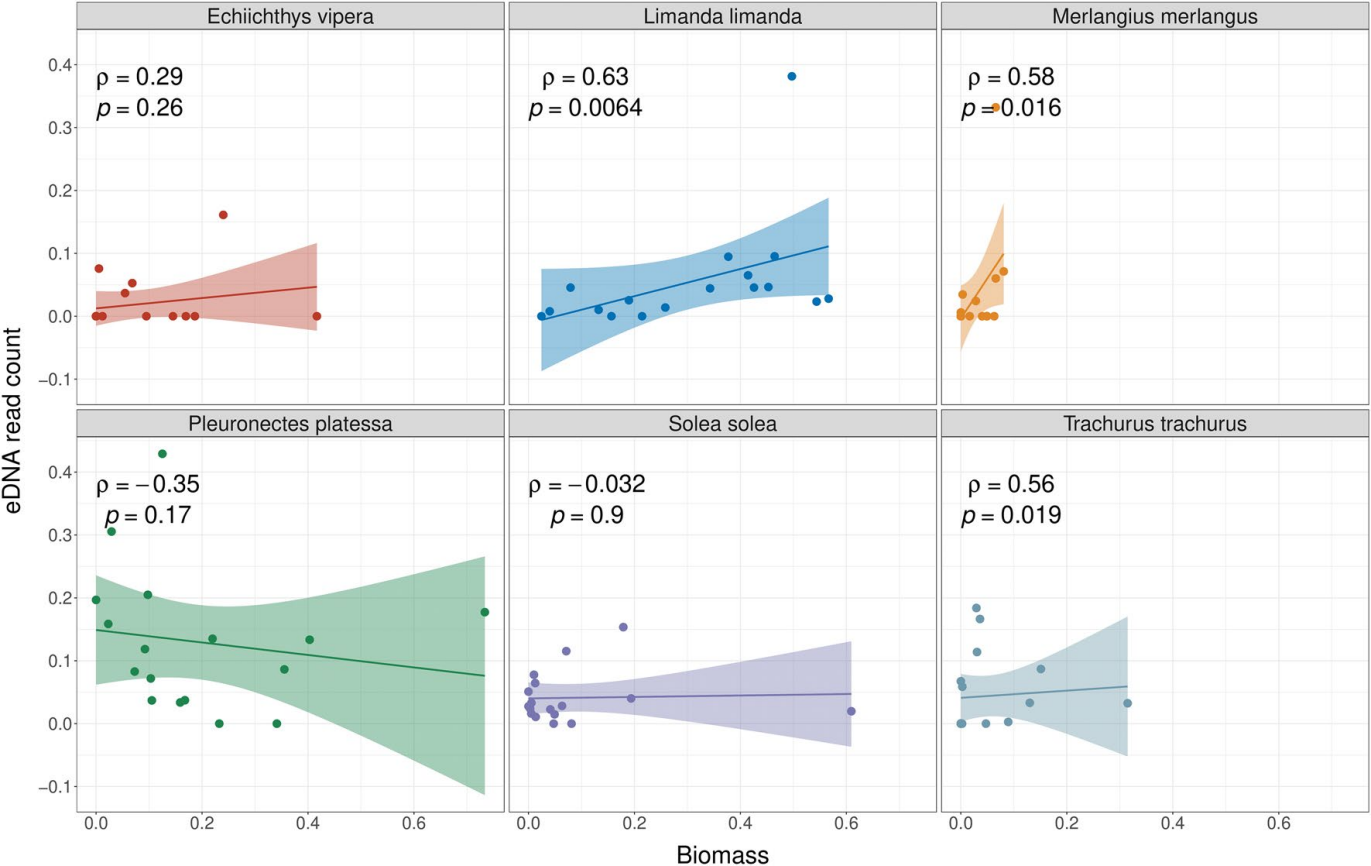
Spearman’s correlation coefficient plots contrasting indexed read counts and relative catch-based biomass. Shaded area around the linear regression line represents the 95% confidence interval.

Spearman’s correlation coefficients were significant between the read abundance and the catch-based biomass/abundance for three of the most common species, specifically *Limanda limanda, Trachurus trachurus* and *Merlangius merlangus.* However, only for *Limanda limanda* a strong correlation between read counts and catch-based abundance (rho = 0.76, p < 0.001) was observed. For *Solea solea, Echiichthys vipera* and *Pleuronectes platessa,* no significant correlation was found between the read count and the catch-based abundance/biomass (Figs. 5 and 6).

## Discussion

In the dynamic and well-mixed waters of the BPNS, no vertical pattern (between surface and bottom samples) in the eDNA signal was distinguished in terms of species richness, Shannon diversity index and community composition. Independent of sample depth, eDNA metabarcoding data successfully discerned the well-known spatial patterns for this area by detecting three distinct fish communities occurring in the coastal, transition and offshore zone. On the other hand, no strong correlation was detected between the eDNA data and catch-based biomass/abundance except for the correlation between read counts and abundance of *Limanda limanda*.

### eDNA metabarcoding detects more fishes than morphological identification of beam trawl catch data

In our study, out of 63 species collectively identified to the species level by eDNA metabarcoding and beam trawling, 34.9% were exclusively identified by eDNA metabarcoding, while only 15.9% were exclusively detected by beam trawling. When eDNA was included in the monitoring effort, the taxonomic detection increased by 53.7% (22 species exclusively detected by eDNA / 41 species detected by beam trawl *100). All the common species native to the study area except *Callionymus lyra* were successfully identified by both method. Even though *C. lyra* was initially detected by eDNA, it was excluded from the dataset after the decontamination step. From those exclusively identified by eDNA metabarcoding, pelagic species had the highest read count (*Sardina pilchardus* and *Scomber Scombrus*), ten species were known to be rare in the study area based on the long-term monitoring data collected between 2008 and 2020 (*Diplecogaster bimaculata, Gasterosteus aculeatus, Scophthalmus rhombus, Dasyatis pastinaca, Pholis gunnelus, Raja brachyura, Taurulus bubalis, Cyclopterus lumpus, Gymnammodytes semisquamatus* and *Aphia minuta*), two species are migrating species (*Anguilla Anguilla,* and *Salmo salar*), four species were never caught with beam trawl nor observed in the area between those years (*Lophius piscatorius, Zeugopterus regius, Salmo salar,* and *Alopias vulpinus*) and one species reported to be non-native in the Belgian waters (*Neogobius melonostomus*)^58^.

Since bottom trawling targets predominantly demersal fish species, pelagic species generally remain out of reach of this method. Approximately 43% of the fish species exclusively detected through eDNA metabarcoding were pelagic/benthopelagic, including widely distributed species such as *Scomber scombrus.* The method also provided valuable information about the distribution of pelagic species. Even though indicator species analysis did not reveal a strong association for *Merlangius merlangus* (indicator value = 0.674), we found a considerable amount of eDNA of the species in coastal locations. In contrast, *Scomber scombrus* and *Trachurus trachurus* demonstrated to be the indicator species of the offshore zone. *Trachurus trachurus* is one of the top ten species with the highest read abundance, with over 65 000 total reads, encompassing the 3% of all marine fish reads (Supplementary Information 2; Total read counts). Despite spawning along the Belgium coast in early summer, the species is known to migrate to southern waters and leave the North Sea from October onwards which coincides with the sampling campaign dates^59^. The high eDNA read abundance of *T. trachurus* is likely caused by this seasonal migration trajectory of the species through the English Channel.

One of the most remarkable identifications in our study was the detection of *Sardina pilchardus* via eDNA metabarcoding, which exhibited the highest read abundance, encompassing 30% of all the marine fish reads among all detected species and was detected in most of the samples, despite not being captured by the beam trawl. Previous studies confirmed the presence of established spawning populations of *S. pilchardus* within the southern North Sea^60^. The spawning period of this warm-water species has been observed to extend until June/July^61^. These findings suggest that juveniles and young adults of *S. pilchardus* may be present in the study area during the sampling campaign.

Of the species exclusively identified by eDNA metabarcoding, 43.5% were classified as rare or uncommon based on the long term monitoring data. *Aphia minuta* and *Pholis gunnelus,* both coastal species and listed as threatened in Dutch waters^62^, were successfully identified through eDNA metabarcoding. Another species, *Raja brachyura,* a near-threatened elasmobranch in the North Sea due to the bycatch of mixed teleost-targeting fisheries^63^ and considered as a Data Limited Stock by the ICES^64,65^ was also detected by eDNA metabarcoding in two locations. One notable genus identified by eDNA metabarcoding is *Alosa* sp*.*, which experienced a pronounced decline in the early 2000s, mostly due to the dam constructions on the river systems and preventing the species reaching their spawning grounds^66^. The eDNA of the species was exclusively detected in coastal locations with low read abundances, aligning with the acoustic telemetry tracking observations conducted by Breine et al.^67^ which showed that *Alosa fallax* individuals were migrating from the Scheldt estuary to the coastal area (and not further offshore) in the BPNS. These results highlight the effectiveness of eDNA metabarcoding in biodiversity monitoring surveys, as it increases the detection rate of species that would otherwise go unnoticed due to their depth preferences and rarity^68,69^.

Traditional morphological identification may encounter limitations when dealing with closely related species as exemplified by *Pomatoschistus* sp. in this study. Despite successfully differentiating two members of this genus, namely *Pomatoschistus minutus and Pomatoschistus lozanai,* morphological identification falls short to identify *Pomatoschistus pictus*, which is a well-known goby species in the study area. eDNA metabarcoding successfully distinguished all three species. Additionally, the long-term beam trawl monitoring data reveals a broad distribution of *Pomatoschistus* sp. spanning from coastal to the offshore zone. eDNA metabarcoding elucidates the species-level distribution, indicating a strong association of *P. minutus* and *P. lozanoi* with coastal waters.

Conversely, the species with identical 12S fragment that remained unidentified via eDNA metabarcoding highlight the necessity of a thorough knowledge of the marker gene’s variability to avoid false negative results. Finally, physical parameters, such as low DNA concentrations of the target species in the water column and the rapid dispersion of eDNA due to water movements are some of the potential reasons that hamper the detection of a species^14^. These parameters are likely why the species*, Spondyliosoma cantharus* and *Osmerus eperlanus,* which had very low biomass and abundance in the beam trawl data and were found in only one location, were not detected in eDNA samples.

### Similar eDNA signals for surface and bottom samples in well-mixed North Sea waters

In terms of resembling hydrographic conditions in the BPNS, the study conducted by Monuki et al.^41^ in a wave-exposed coastal site with a maximum depth of 10 m stands out. The location experienced high daily tidal fluctuations and strong currents, promoting vertical mixing. The study demonstrated a vertical pattern in eDNA distribution that correlated with the depth preferences of the species (demersal vs. pelagic). Conversely, we did not observe any distinct vertical eDNA signals in terms of diversity metrics or community structure analysis, as visualized by the NMDS plot. In our study, the eDNA signals solely captured by surface samples belongs to *Cyclopterus lumpus, Diplecogaster bimaculata, Gasterosteus aculeatus, Lophius piscatorius, Pomatoschistus pictus*, and *Zeugopterus regius*. These species are all demersal or benthopelagic and only detected in one or two samples with low eDNA abundances. Additionally, the eDNA of pelagic species such as *Sardina pilchardus, Scomber scombrus* and *Engraulis encrasicolus* was successfully captured in bottom samples, further supporting the absence of a discernable vertical eDNA pattern reflecting the depth preferences of the species. This result shows the significant influence of mixing on eDNA patterns which facilitates the dispersion of eDNA vertically throughout the water column in the BPNS.

In our study, alpha and beta diversity patterns were highly similar between the two depths. In the context of biodiversity assessment, sampling in one depth has demonstrated its adequacy in capturing the majority of fishes present in the BPNS, including commercially important species such as *S. solea, P. platessa, M. merlangus* and *S. scombrus.* For studies aiming to capture the full spectrum of biodiversity, including rare species that are challenging to detect through bottom trawling, increasing the number of replicates/sampling size from the same sampling depth may facilitate the detection of additional species better than sampling at two depths.

### eDNA metabarcoding reflects known spatial distribution patterns of demersal fish communities

Beta diversity analysis of eDNA metabarcoding data yielded robust discrimination of fish communities across three distinct zones, providing evidence for the presence of a discernable spatial pattern within the BPNS^40^, demonstrating strong compatibility with long-term monitoring. Indicator species analysis for eDNA metabarcoding and simultaneous beam trawl catch data showed high similarity for the coastal zone, whereas eDNA metabarcoding data revealed a higher number of indicator species associated with the offshore zone than the indicator species detected by beam trawl catch data, partly due to the fact that eDNA metabarcoding provides more information about pelagic species than beam trawl catch data.

eDNA metabarcoding revealed the widespread distribution of *Solea solea,* and *Pleuronectes platessa* across all three zones in the BPNS. This finding is largely consistent with the species distribution documented in the area, with the exception of *S. solea*^39^*.* We detected the eDNA of *S. solea* across three zones in the BPNS, while the species was primarily reported to be associated with coastal habitats in beam trawl long term monitoring data. Aggregating fish species, such as *S. solea*, may display temporal small-scale variability^70^. *Solea solea* migrate to warmer offshore waters in autumn and form dense aggregations in the southern North Sea and eastern English Channel^71^. Additionally, adults from different nursery grounds may also move to their southern spawning grounds through the English Channel from September to February^72^. These migratory movements may explain the relatively high detection of *Solea solea* with eDNA metabarcoding compared to the beam trawl catch data. *Mullus surmelutus* and *Echiichthys vipera*, two of the most dominant species of the offshore zone based on long-term monitoring^39^, emerged as indicator species of the offshore zone in the eDNA metabarcoding data, but not in the simultaneous trawl data. This could be attributed to the aggregating behavior of the species, resulting in a patchy distribution that hampers detection by trawling^73^. In summary, eDNA metabarcoding successfully reproduced the distribution of the communities obtained from long-term monitoring and offered a more comprehensive assessment of the indicator species of the communities compared to the trawl data.

### The power of eDNA metabarcoding to quantify fish abundance or biomass remains dubious

The quantitative assessment of fish communities via eDNA metabarcoding is an ongoing debate in metabarcoding research. Some studies have confirmed the positive linear correlation between read counts and fish abundance/biomass in marine environments^14,74–76^, while others have shown a lack of significant relationship between read abundance and catch-based abundance/biomass^32,35^. In our study, we observed a strong correlation between read abundance and catch abundance only for *Limanda limanda. Trachurus trachurus* and *Merlangius merlangus* returned moderate correlation coefficients or high variability, despite significant p-values. Conversely, the read counts of *Echiichthys vipera*, *Solea solea* and *Pleurenectes platessa,* did not exhibit any significant correlation with catch abundance or biomass metrics.

The biotic and abiotic factors influencing the eDNA concentrations in marine ecosystems are likely to influence the quantitative correlation of eDNA abundance with catch-based biomass and abundance. From those factors, the eDNA shedding rate may be correlated to the age, sex, diet, season, biomass, and surface area of the organism^33^. Moreover, taking into account that 22 mm mesh size cod-end targets juveniles and young adults rather than the commercial size individuals, the catch-based abundance/biomass data may not be the accurate representation of the fish populations. Another important factor to consider is amplification bias. Species with high initial DNA concentrations are likely to experience higher PCR amplification, skewing the read abundance in favor of the most abundant species^77^. Variations in primer-template binding efficiency may also influence the read abundance. In a mixed DNA template pool, certain species may exhibit enhanced amplification, while others may experience reduced amplification or fail to amplify altogether due to variations in the primer binding sites^78^. Considering this, it is crucial to exercise caution while interpreting the eDNA metabarcoding read abundance as a proxy of actual quantitative metrics.

## Conclusion

Our study demonstrates the efficacy of eDNA metabarcoding in reproducing the fish community patterns that are highly compatible with the long term beam trawl data within the BPNS. While a few species persisted in being exclusively identified through trawl catch data, the utilization of eDNA metabarcoding significantly contributed to the monitoring effort, increasing the detection of the taxonomic diversity by 53.7%. Moreover, we showed that shallow and well-mixed waters of the BPNS do not exhibit distinct vertical eDNA patterns corresponding to the depth preferences of the fish species. Sampling at different depths does not significantly affect the alpha and beta diversity within the area, hence sampling at one depth should be sufficient to capture the majority of the fish diversity. Even though the method cannot yet replace the catch-based surveys in providing robust quantitative estimates, integration of eDNA analyses in monitoring efforts may substantially contribute to our understanding of biodiversity and community composition of the fish, ultimately leading to more effective and comprehensive management strategies for marine ecosystems.

### Supplementary Information

Supplementary Information 1.

Supplementary Information 2.

Supplementary Information 3.

Supplementary Information 4.

## Data Availability

The sequencing data and corresponding metadata generated for this study are available on the online NCBI repository (BioProject number: PRJNA1032405) and on the GBIF database (10.15468/3ygzac). The scripts and the custom reference database are available on the Github platform (https://github.com/ndukann/NJ2022_eDNA_Metabarcoding).
