# Supplementary material for: Vertical and horizontal environmental DNA (eDNA) patterns of fish in a shallow and well-mixed North Sea area
Source: Sci Rep. 2024 Jul 20;14:16748. doi: 10.1038/s41598-024-66912-2 (PMC11271445; doi:10.1038/s41598-024-66912-2)
Supplement: Supplementary file 4 — Supplementary Information 4. [file 41598_2024_66912_MOESM4_ESM.pdf]

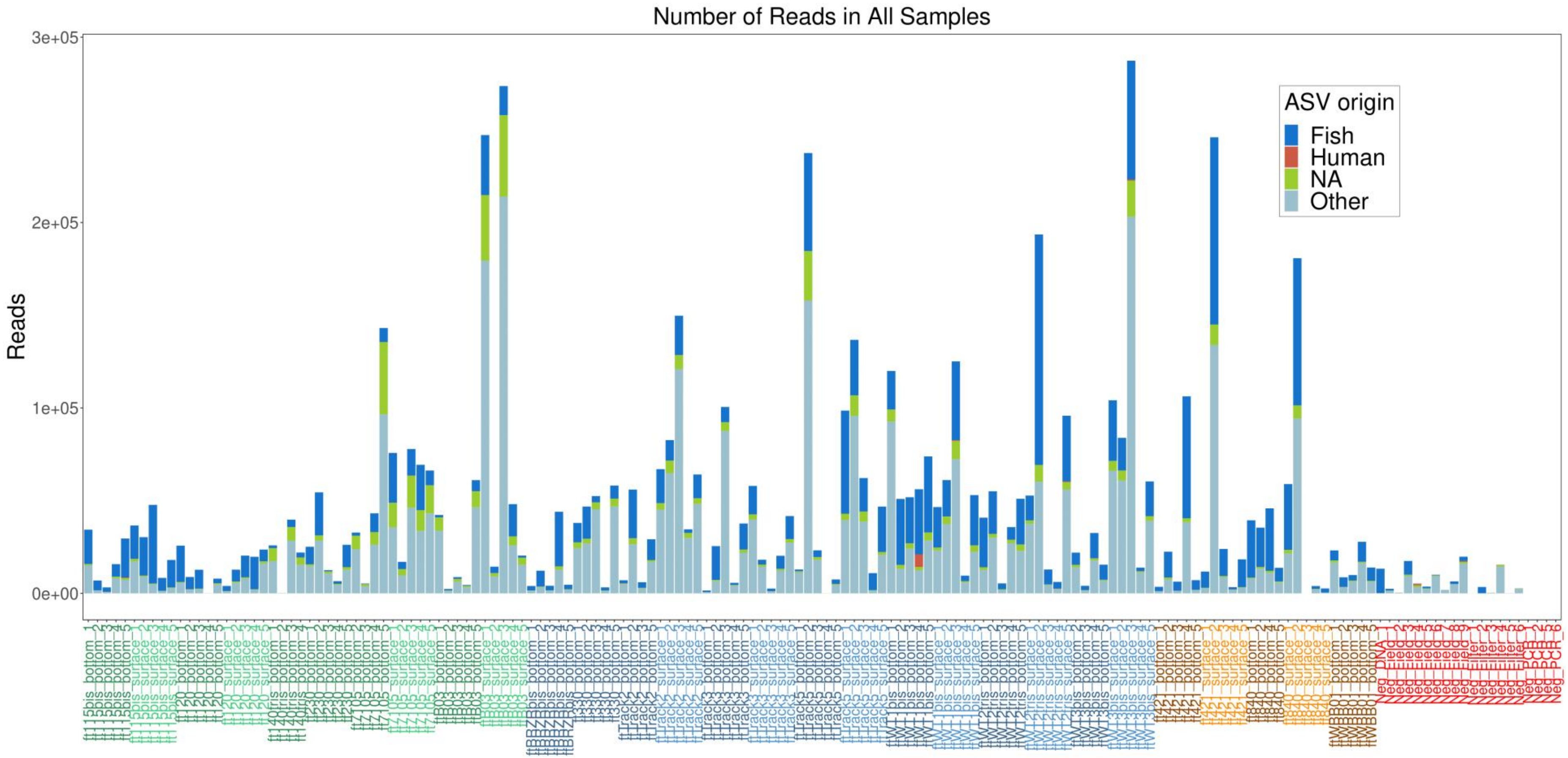

Figure S1: Number of reads per sample after DADA2 (unrarefied data). The sample names on the x-axis were colored according to the sampling zone and depth of the eDNA water samples: Coastal surface (light green), coastal bottom (dark green), transition surface (light blue), transition bottom (dark blue), offshore surface (light orange), offshore bottom (dark orange) and negative control samples (red).

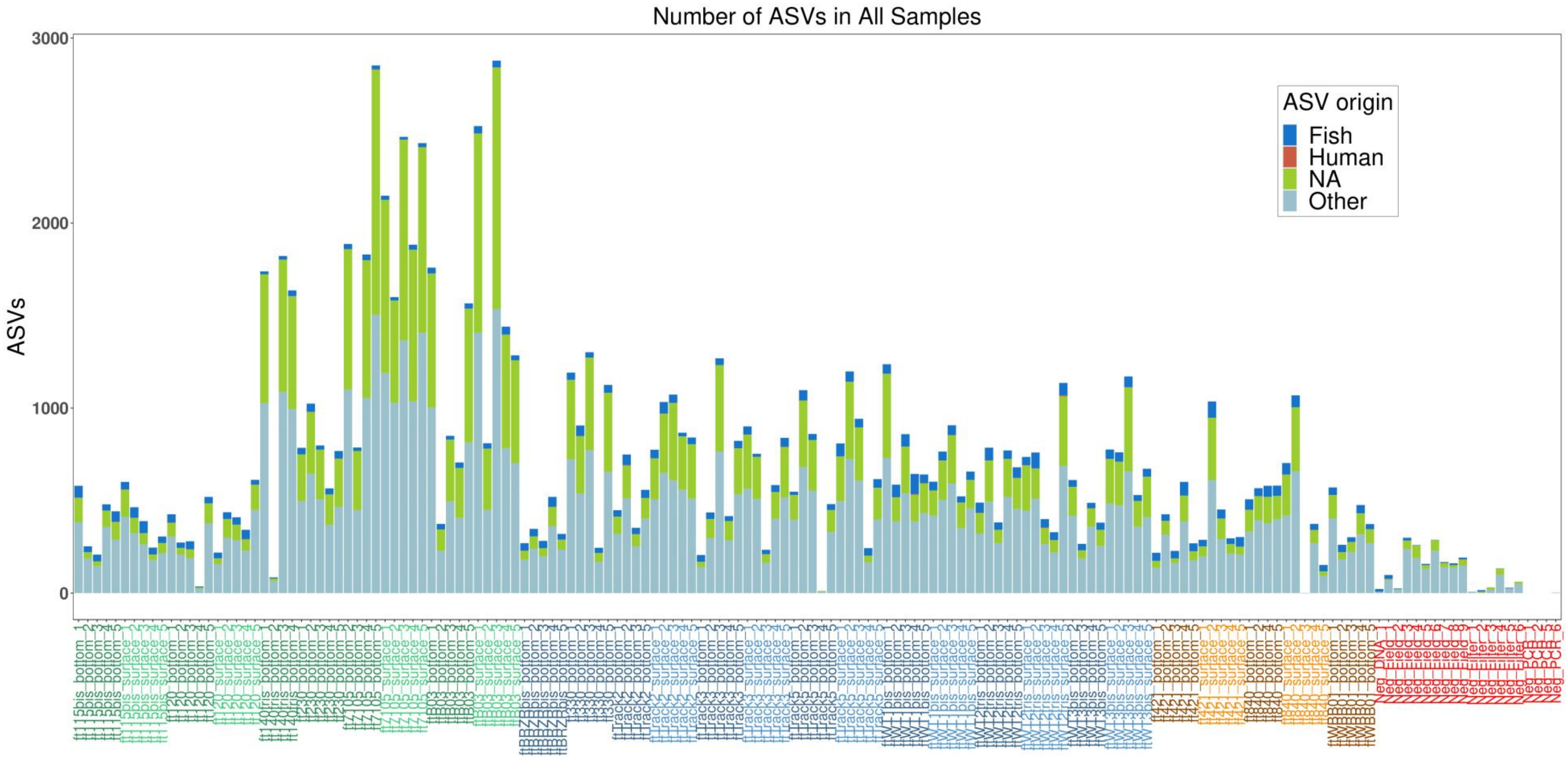

Figure S2: Number of ASVs per sample after DADA2 (unrarefied data). The sample names on the x-axis were colored according to the sampling zone and depth of the eDNA water samples: Coastal surface (light green), coastal bottom (dark green), transition surface (light blue), transition bottom (dark blue), offshore surface (light orange), offshore bottom (dark orange) and negative control samples (red).

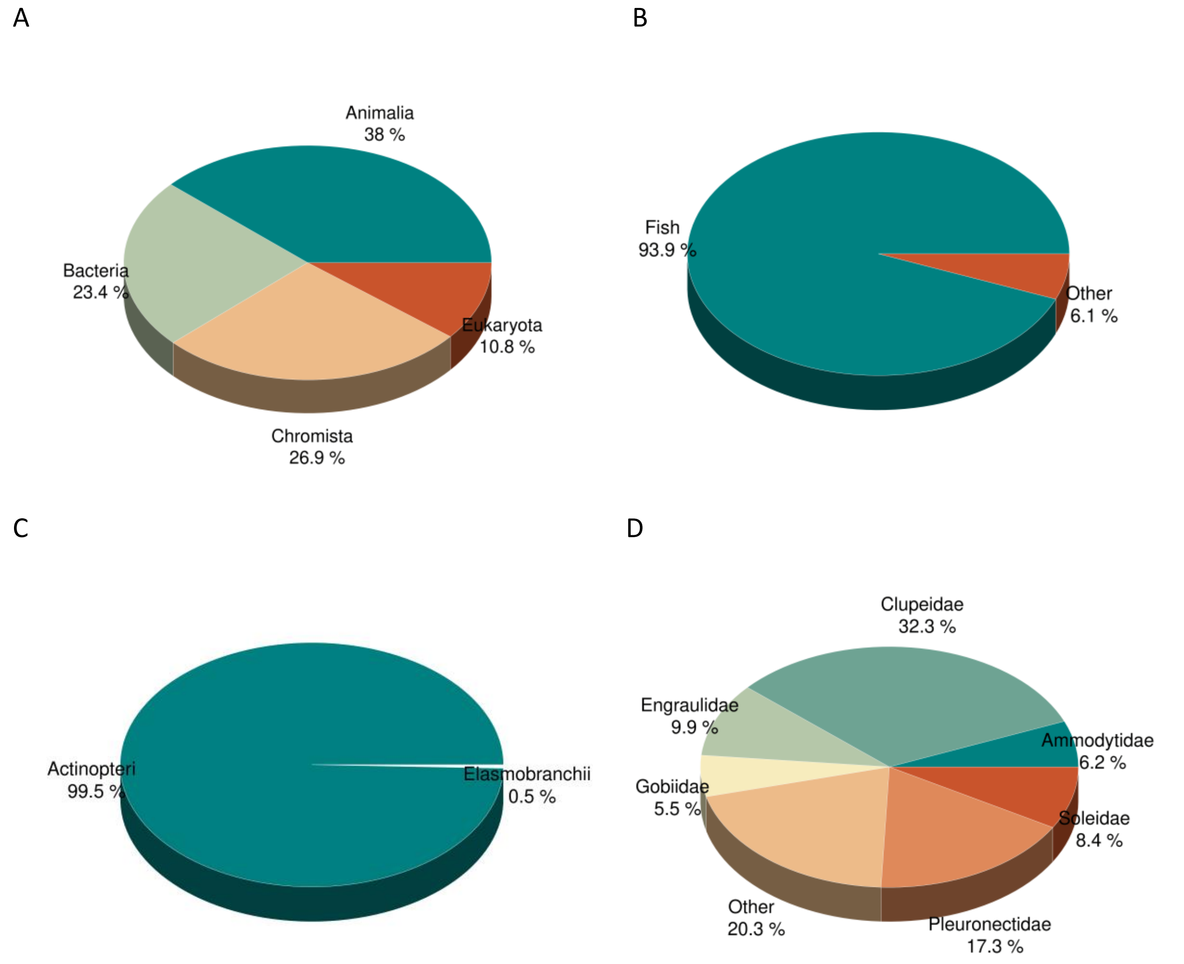

Figure S3: Percentage distribution of sequence reads of unrarefied data among A) three kingdoms (Plantae, Fungi and Protista kingdoms are excluded due to less than 1% representation) and Eukaryota domain which were not assign to kingdom-level, B) Animalia kingdom, C) two target fish classes; Actinopteri and Elasmobranchii (Chondrichthyes was also gathered under Elasmobranchii) , D) Actinopteri families.

A

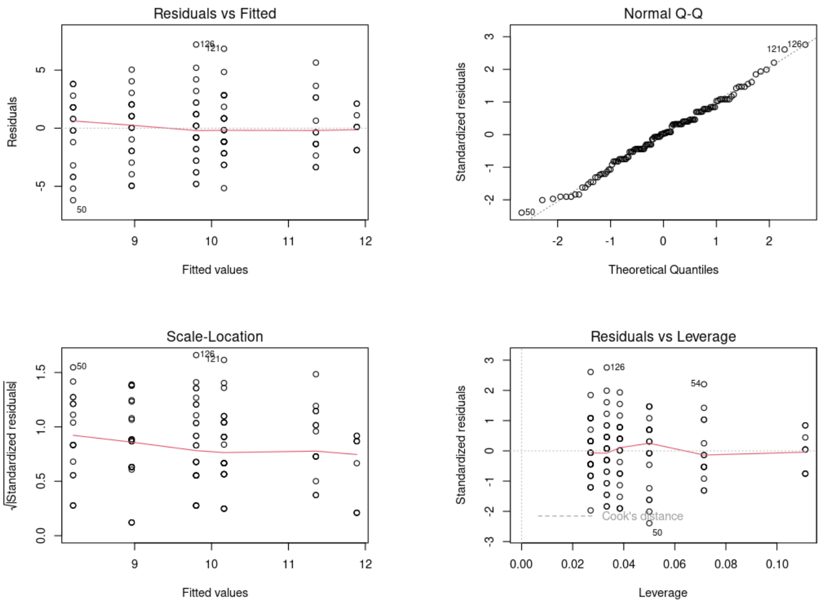

B

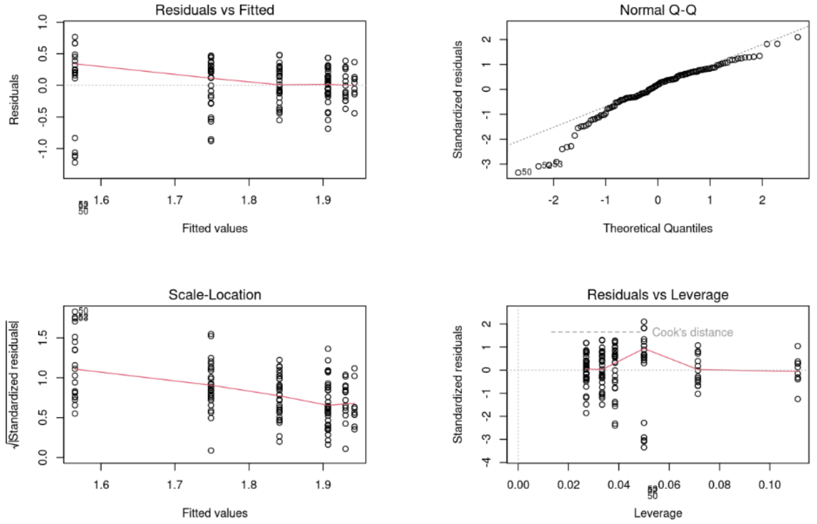

Figure S4: QQ Plots of A) species richness B) Shannon diversity index models constructed using the main factors depth and zone, as well as their interactions (zone*depth).

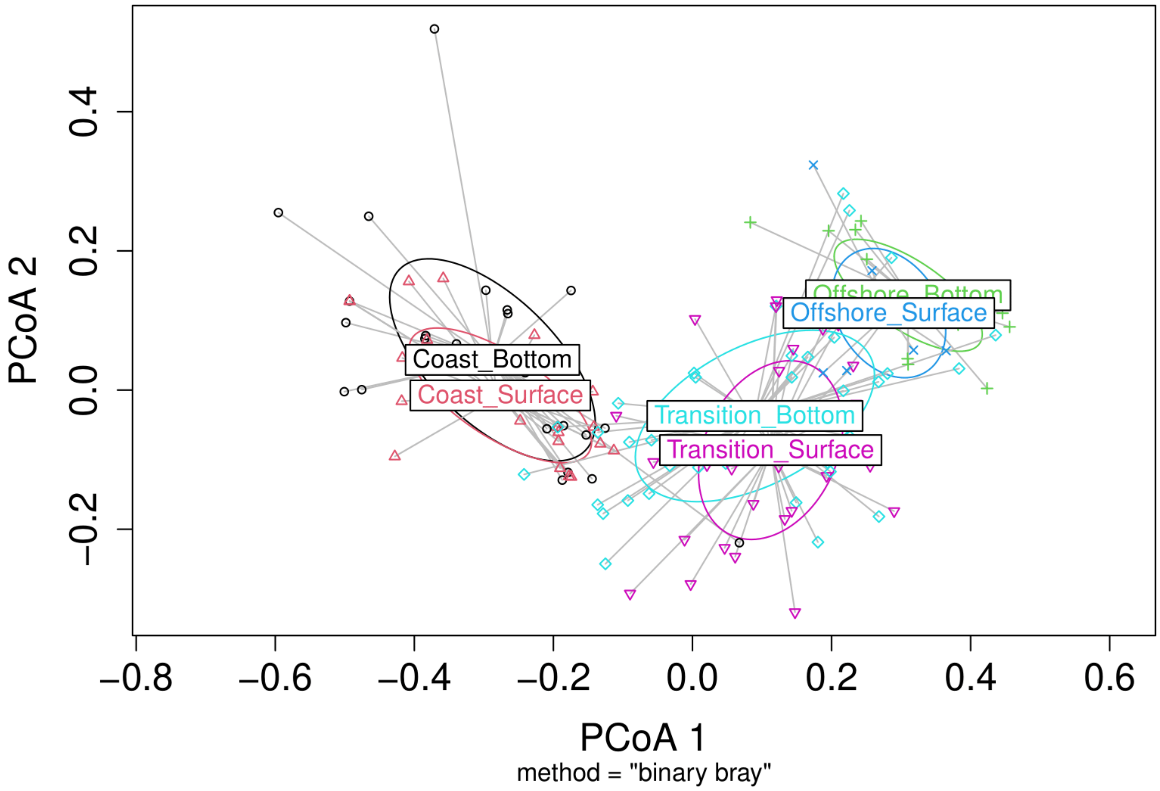

Figure S5: The BETADISPER plot with 9999 permutations and centroid dispersion type. Groups are defined as the interaction of each zone and depth.

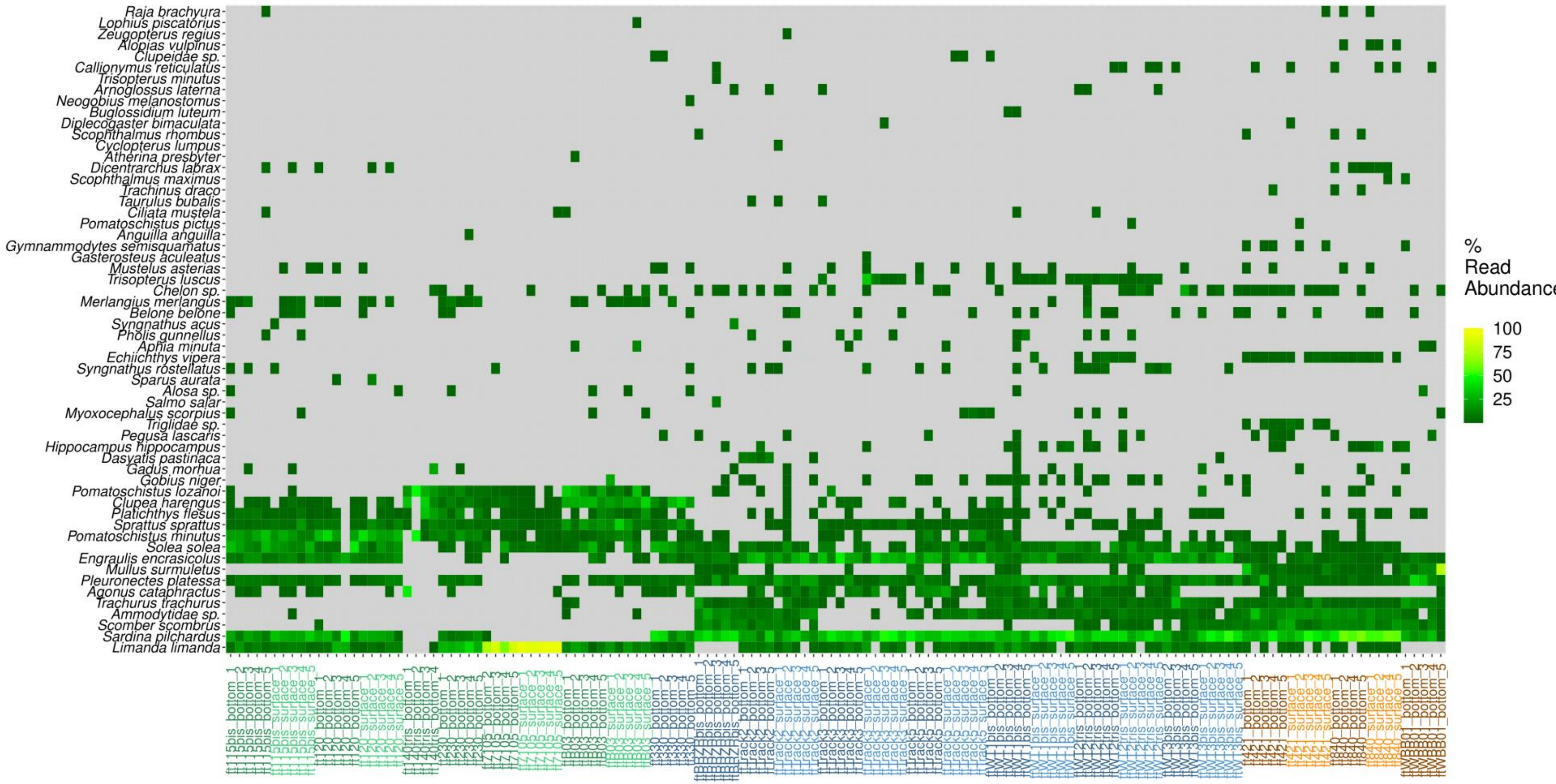

Figure S6: Heat map for the relative read abundance of fish species after rarefaction, before removing the samples with sequencing depth less than 10 000 reads. The sample names on the x-axis were colored according to the sampling zone and depth of the eDNA water samples: Coastal surface (light green), coastal bottom (dark green), transition surface (light blue), transition bottom (dark blue), offshore surface (light orange), offshore bottom (dark orange) and negative control samples (red).

Species richness (S)				
	Df	SumOfSq	F-value	p-value
Depth	1	4.10	0.58	0.45
Zone	2	136.09	9.63	0.00013
Depth:Zone	2	6.18	0.44	0.65
Shannon diversity index (H’)				
Depth	1	-	1.57	0.21
Zone	2	-	0.98	0.38
Depth:Zone	2	-	0.60	0.55

Table S1: Two-way Analysis of Variance (ANOVA) results of alpha diversity indices.

Species richness (S)				
	Mean diff	lwr	upr	p-adjusted
Transition-Coast	1.37	1.17	2.57	0.021
Offshore-Coast	2.93	1.34	4.53	0.00008
Offshore-Transition	1.57	0.05	3.08	0.041

Table S2: Post-hoc Tukey HSD test for the species richness of the zone factor.

	Df	SumOfSqs	R^2	F-value	p-value
Depth	1	0.21	0.011	2.56	0.497
Zone	2	7.44	0.414	46.98	0.0001
Depth:Zone	2	0.03	0.002	0.21	0.987
Residual	132	10.45	0.577		
Total	137	1.00	1.000		

Table S3: Results of PERMANOVA (Permutational Multivariate Analysis of Variance) applied to indexed eDNA community composition distance matrix (Bray-Curtis) for the analysis of species composition based on zone and depth factor (VEGAN package in R, “adonis2” function, 9999 permutations).

	Df	SumOfSqs	MeanSqs	R^2	F-value	p-value
Coast vs Transition	1	4.38	4.38	0.32	53	0.0001
Coast vs Offshore	1	5.53	5.53	0.53	77.46	0.0001
Transition vs Offshore	1	1.47	1.47	0.17	18.35	0.0001

Table S4: Pairwise PERMANOVA results showing the statistical significance of eDNA community composition (Bray-Curtis distance matrix of indexed eDNA data) differences between environmental zones (PAIRWISE package in R, “pairwise.adonis2” function, 9999 permutations).

```
Group Coast  #sps.  8
                            A      B  stat p.value
Platichthys flesus     0.9720 0.8542 0.911  0.0001 ***
Sprattus sprattus      0.8479 0.9583 0.901  0.0001 ***
Pomatoschistus minutus 0.8981 0.8958 0.897  0.0001 ***
Limanda limanda        0.8049 0.9375 0.869  0.0001 ***
Clupea harengus        0.7885 0.8333 0.811  0.0001 ***
Pomatoschistus lozanoi 0.9024 0.5833 0.726  0.0001 ***
Merlangius merlangus   0.9075 0.5000 0.674  0.0001 ***
Solea solea            0.5303 0.8125 0.656  0.0074 **

 Group Offshore  #sps.  17
                                  A       B  stat p.value
Mullus surmuletus           0.94817 0.95652 0.952  0.0001 ***
Scomber scombrus            0.78512 1.00000 0.886  0.0001 ***
Ammodytidae sp.             0.69790 0.95652 0.817  0.0001 ***
Trachurus trachurus         0.62918 1.00000 0.793  0.0001 ***
Echiichthys vipera          0.72018 0.69565 0.708  0.0001 ***
Sardina pilchardus          0.47450 1.00000 0.689  0.0001 ***
Triglidae sp.               1.00000 0.34783 0.590  0.0001 ***
Gymnammodytes semisquamatus 1.00000 0.26087 0.511  0.0001 ***
Hippocampus hippocampus     0.69856 0.34783 0.493  0.0036 **
Dicentrarchus labrax        0.79163 0.26087 0.454  0.0017 **
Pegusa lascaris             0.67061 0.26087 0.418  0.0093 **
Alopias vulpinus            1.00000 0.17391 0.417  0.0010 ***
Callionymus reticulatus     0.59843 0.26087 0.395  0.0158 *
Trachinus draco             1.00000 0.13043 0.361  0.0043 **
Scophthalmus rhombus        0.97618 0.13043 0.357  0.0021 **
Raja brachyura              0.92728 0.13043 0.348  0.0109 *
Scophthalmus maximus        1.00000 0.08696 0.295  0.0265 *

 Group Transition  #sps.  7
                            A      B  stat p.value
Engraulis encrasicolus 0.6483 0.9851 0.799  0.0001 ***
Agonus cataphractus    0.7022 0.6567 0.679  0.0002 ***
Pleuronectes platessa  0.4174 0.9851 0.641  0.0223 *
Trisopterus luscus     1.0000 0.3582 0.599  0.0002 ***
Syngnathus rostellatus 0.8268 0.2537 0.458  0.0103 *
Pholis gunnellus       0.8972 0.1343 0.347  0.0434 *
Dasyatis pastinaca     1.0000 0.1045 0.323  0.0362 *
```

Table S5: Indicator Species Analysis results for eDNA metabarcoding. Component A (specificity) represents the estimated probability of how well the presence of the species predicts that a given location belongs to a specific zone. Component B (fidelity) indicates the probability of the occurrence of the species as an indicator of that zone. Stat value represents the indicator value index produced by those two components.

Group Coast #sps. 7

	A	B	stat	p.value	
Merlangius merlangus	0.9120	1.0000	0.955	0.0007	***
Solea solea	0.9058	1.0000	0.952	0.0051	**
Sprattus sprattus	0.9937	0.8333	0.910	0.0153	*
Clupea harengus	0.9614	0.8333	0.895	0.0080	**
Limanda limanda	0.7210	1.0000	0.849	0.0125	*
Myoxocephalus scorpius	1.0000	0.6667	0.816	0.0125	*
Platichthys flesus	1.0000	0.6667	0.816	0.0127	*

Group Offshore #sps. 1

	A	B	stat	p.value	
Pegusa lascaris	0.8309	0.6667	0.744	0.0371	*

Table S6: Indicator Species Analysis results for morphological identification. Component A (specificity) represents the estimated probability of how well the presence of the species predicts a given location belongs to a specific zone. Component B (fidelity) indicates the probability of the occurrence of the species as an indicator of that zone. Stat value represents the indicator value index produced by those two components.
